# Silver-Coated Distal Femur Megaprosthesis in Chronic Infections with Severe Bone Loss: A Multicentre Case Series

**DOI:** 10.3390/jcm12206679

**Published:** 2023-10-23

**Authors:** Michele Fiore, Andrea Sambri, Lorenzo Morante, Marta Bortoli, Stefania Claudia Parisi, Francesco Panzavolta, Domenico Alesi, Elisabetta Neri, Maria Pia Neri, Sara Tedeschi, Eleonora Zamparini, Luca Cevolani, Davide Maria Donati, Pierluigi Viale, Domenico Andrea Campanacci, Stefano Zaffagnini, Massimiliano De Paolis

**Affiliations:** 1 Department of Medical and Surgical Sciences, Alma Mater Studiorum University of Bologna, 40138 Bologna, Italy; michelefiore.md@gmail.com (M.F.); sara.tedeschi5@unibo.it (S.T.); pierluigi.viale@unibo.it (P.V.); 2 Orthopaedics and Traumatology Unit, IRCCS Azienda Ospedaliero-Universitaria di Bologna, 40138 Bologna, Italy; lorenzo.morante@ior.it (L.M.); marta.bortoli@ior.it (M.B.); stefaniaclaudia.parisi@ior.it (S.C.P.); francesco.panzavolta@studio.unibo.it (F.P.); massimiliano.depaolis@aosp.bo.it (M.D.P.); 3 Second Orthopaedic Clinic, IRCCS Istituto Ortopedico Rizzoli, 40136 Bologna, Italy; domenico.alesi@ior.it (D.A.); mariapia.neri@ior.it (M.P.N.); stefano.zaffagnini@ior.it (S.Z.); 4 Orthopaedic Oncology Unit, Azienda Ospedaliera Universitaria Careggi, 50134 Firenze, Italy; neriel@aou-careggi.toscana.it (E.N.); domenicoandrea.campanacci@unifi.it (D.A.C.); 5 Infectious Disease Unit, Department for Integrated Infectious Risk Management, IRCCS Azienda Ospedaliero-Universitaria di Bologna, 40138 Bologna, Italy; eleonora.zamparini@aosp.bo.it; 6 Third Orthopaedic Clinic, IRCCS Istituto Ortopedico Rizzoli, 40136 Bologna, Italy; luca.cevolani@ior.it (L.C.); davidemaria.donati@ior.it (D.M.D.)

**Keywords:** orthopedic surgery, megaprosthesis, distal femur, silver coating, periprosthetic joint infections, fracture-related infections, bone loss

## Abstract

Periprosthetic joint infections (PJI) and fracture-related infections (FRI) of the distal femur (DF) may result in massive bone defects. Treatment options include articulated silver-coated (SC) megaprosthesis (MP) in the context of a two-stage protocol. However, there is limited evidence in the literature on this topic. A retrospective review of the prospectively maintained databases of three Institutions was performed. Forty-five patients were included. The mean follow-up time was 43 ± 17.1 months. Eight (17.8%) patients had a recurrent infection. The estimated recurrence-free survival rate was 91.1% (93.5% PJI vs. 85.7% FRI) 2 years following MP implantation, and 75.7% (83.2% PJI vs. 64.3% FRI; *p* = 0.253) after 5 years. No statistically relevant difference was found according to the initial diagnosis (PJI vs. FRI). Among possible risk factors, only resection length was found to significantly worsen the outcomes in terms of infection control (*p* = 0.031). A total of eight complications not related to infection were found after reimplantation, but only five of them required further surgery. Above-the-knee amputation was performed in two cases (4.4%), both for reinfection. Articulated DF SC MP in a two-stage protocol is a safe and effective treatment for chronic knee infection with severe bone loss.

## 1. Introduction

Reconstruction of massive defects of long bones is a demanding surgical procedure that poses multiple challenges for the treating orthopedic surgeon [1,2]. Several clinical scenarios may be associated with significant bone loss, which are comparable to the resection of a bone tumor. These may include severe trauma, periprosthetic fractures, failed osteosynthesis with a non-union, fracture-related infection (FRI) with chronic osteomyelitis, multiple revision of arthroplasty for either an aseptic loosening, or a periprosthetic joint infection (PJI) [3,4,5,6,7,8,9,10,11,12]. Patients presenting any of the above are likely to have undergone multiple previous procedures which may limit the options of reconstruction or may present several comorbidities.

There are various reconstructive strategies to treat bone defects such as autograft and allogeneic bone grafting, bone transport, the use of standard revision prosthesis, and megaprosthesis (MP) [1,13,14,15,16]. The use of MP, previously reserved for oncological reconstructions, has gradually been extended to non-oncological reconstructions [6].

Thanks to its intraoperative versatility, MP allows for the reconstruction of large bone defects, even at the joint level, with the possibility of limb-sparing surgery and the preservation of joint motion, as well as the predictable and usually prompt recovery after surgery [17,18]. In fact, MP is often the preferable treatment option, particularly in elderly patients or in patients requiring short length of stay and a rapid recovery because of low activity levels and multiple comorbidities [18,19].

However, MP has inherent disadvantages including implant costs, limited further revision options, and PJI [2,20]. Infections are reported in up to 50% of oncological cases [21] and 0–44% of non-oncological cases, with a significant discrepancy depending on the etiology [6,21]. Among the non-oncological indications, cases treated for previous infections are at higher risk of PJI [6].

Therefore, in order to reduce the incidence of PJI, antibacterial surfaces whose antimicrobial properties are associated with low human toxicity have been marketed [21], and many of them are currently undergoing preclinical studies [22,23,24]. Of these, silver-coated (SC) surfaces have proved to be the most effective and safe, due to the broad spectrum of antibacterial properties of the silver, low bacterial resistance, and low cytotoxicity [21]. Although the utility of silver coating in preventing infection is uncertain following primary surgery, there is relevant available evidence suggesting that silver coating in oncological and non-oncological scenarios reduces the infection rate after MP implantation in revision surgeries, especially when treating previous infections [21].

Indeed, in infectious contexts where MP may be a treatment option, bone and soft tissue (ST) conditions are radically different from those of the oncologic patient. As for the bone, it is frequently affected by chronic osteomyelitis with necrotic areas, or it is osteoporotic and distorted by previous prostheses or other hardware; moreover, septic non-unions are common in post-traumatic cases. Regarding ST impairment, this is particularly relevant for the knee. The extensor mechanism is often critical, particularly in septic post-traumatic patients or in patients with PJI recurrences who have undergone multiple surgeries [25,26]. Tissue adhesion, scar interference, muscular and tendon impairment, ST retractions, and skin problems can lead to a reduced function of the knee and/or severe joint stiffness, creating adverse conditions during the reconstructive step [27]. These scenarios often require a combination of plastic surgery procedures to ensure adequate coverage of the implant, as well as to improve local blood circulation to allow antibiotics to diffuse and improve the immune response of host tissues [28].

The key aspect of treatment is thorough debridement of infected bone and soft tissues. The extent of debridement often poses the question of how to manage the bone deficit, as well as the residual dead space. A staged revision is a suitable option for the treatment of chronic joint infections with sever bone loss and possible ST impairment. The first stage includes removal of the hardware and debridement of bone and soft tissues, including bone resection if needed, as well as concomitant treatment with a microbe-specific antibiotic. Implantation of a temporary antibiotic-loaded cement spacer, performed at the time of prosthesis removal, is mandatory in case of severe bone loss at the distal femur (DF). The second stage consists of MP implantation, when clinical assessment and laboratory tests do not suggest a residual infection.

The aim of this retrospective study is to describe the mid-term results of infection control in the treatment of total knee arthroplasty (TKA) PJI and DF FRI with severe bone deficiency using SC articulated DF MP, in the context of a two-stage treatment.

## 2. Materials and Methods

**Figure 1:**
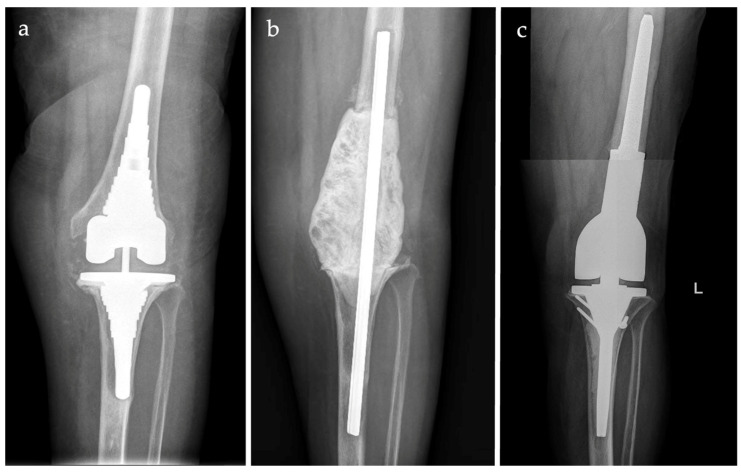
Case of distal femur silver-coated megaprosthesis implantation for periprosthetic joint infection (PJI). Distal femur osteomyelitis with septic loosening of revision knee prosthesis implanted after previous two-stage treatment for PJI (**a**). Antibiotic-loaded cemented spacer after first-stage (**b**). Cemented megaprosthesis; tibial tubercle osteotomy (then fixed with screws) was performed to facilitate implant placement considering the severe stiffness after the first-stage procedure (**c**).

**Figure 2:**
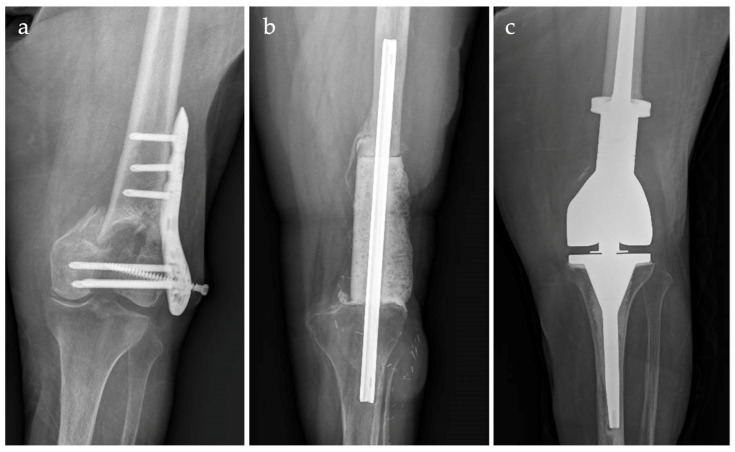
Case of distal femur silver-coated megaprosthesis implantation for fracture-related infection. Septic non-union after open reduction and internal fixation for a distal femur fracture (**a**). Antibiotic-loaded cemented spacer after first-stage; soft tissue injury required a gastrocnemius rotation flap (**b**). Cemented megaprosthesis (**c**).

A retrospective review of the prospectively maintained databases of three Institutions was undertaken for all patients with an initial diagnosis of distal femur (DF) chronic periprosthetic joint infection (PJI) of a standard or revision total knee arthroplasty (TKA) (Figure 1a–c) or DF fracture-related infection (FRI) (Figure 2a–c) treated between 2013 and 2021 with a silver-coated (SC) DF megaprosthesis (MP) after a staged approach. Only patients with a severe bone stock deficiency (grade 3, according to the AORI classification) after first-stage were included.

Only patients with complete clinical, radiological, and microbiological data, and at least 24 months of follow-up and patients who provided informed consent were included.

Prosthetic joint infection was clinically confirmed according to criteria defined by the Musculoskeletal Infection Society (MSIS) [29]. Diagnosis of FRI was made according to criteria defined in FRI consensus definition [30,31].

Each patient’s clinical status was assessed using the American Society of Anesthesiologists (ASA) score and the Charlson Comorbidity Index (CCI). Smoking habits, alcoholic habits, diabetes, and body mass index (BMI) at baseline were also recorded.

**Figure 3:**
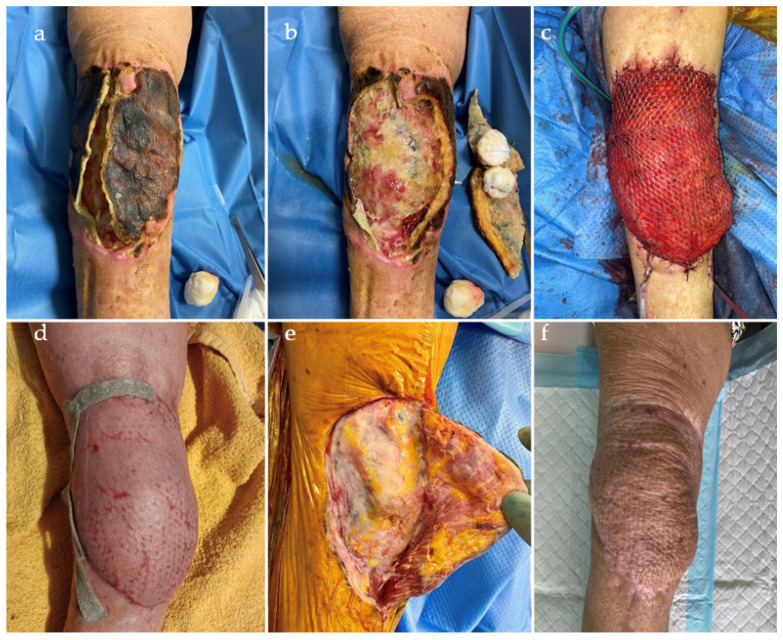
Case of distal femur silver-coated megaprosthesis implantation for periprosthetic joint infection after total knee replacement, complicated by skin necrosis (**a**). Debridement and negative pressure wound therapy (**b**). *Latissimus dorsi* flap with geniculate vessels anastomosis and Tiersche during first stage (**c**). Flap healing after interstage time (**d**). Surgical access for second stage on previous flap, preserving vascular pedicle (**e**). Result after second stage (**f**).

All patients were treated with a staged approach. In details, at the first stage, considering the bone loss and incompetence of the knee stabilizers, a temporary arthrodesis was conducted, using a static, intramedullary spacer, composed of metal rods, covered with antibiotic-loaded polymethylmethacrylate (PMMA). The prosthesis or different hardware was sent for sonication or treatment with dithiothreitol each time [32]. All infected or necrotic bone was removed, and the soft tissue (ST) was circumferentially debrided. In all cases, bone resection of the distal femur was performed. Specimens were taken from representative areas to perform antibiotic susceptibility test (AST). The full MSIS/FRI criteria for infection were met in all patients. In the case of a fistula, the sinus tract was always completely excised, and the tissue sent for histological examination. At the time of the first stage, in the case of severe ST injuries, orthoplastic coverage procedures were associated: rotational gastrocnemius flap or vascularized free flap (anterolateral thigh perforator flap or *latissimus dorsi* free flap, with both arterial and venous anastomosis to the geniculate vessels), depending on the characteristics of the individual case (Figure 3a–f).

All patients were discussed at a multidisciplinary infection board [33], and suitable systemic antibiotic therapy was given according to AST for 4–6 weeks.

Each spacer was maintained for at least 6 weeks. If there was still clinical evidence of persistent infection, then the patient underwent repeat debridement and spacer exchange, and a new course of systemic antibiotics was administered. If clinical findings and laboratory results did not suggest a residual infection, the patient underwent the second stage. At the second stage, an articulated MP (Megasystem C^®^, Waldemar Link GmbH & Co. KG, Hamburg, Germany) with silver-coating (PorAg^®^, Waldemar Link GmbH & Co. KG, Hamburg, Germany) was implanted in all patients included in this series. In case of severe stiffness, a tibial tubercle osteotomy was performed in order to facilitate the removal of eventual previous cemented tibial stem, implanted during the first stage, or to facilitate implant placement during the second stage, then fixed with three divergent screws both at the first or second stage [34]. Stems cementation was performed according to bone quality and patient age.

Patients were routinely evaluated with knee X-rays and blood C-reactive protein (CRP) monthly during the first 6 months and every 3 months up to the second year after surgery. Successful eradication of the infection was defined according to Fillingham et al. [35]. Infection control status after surgery was the main outcome. The term “reinfection” will be applied to both plausible recurrences and possible different infections. Reinfection was defined as sinus formation, additional surgery infection, or the need for chronic antibiotic treatment for persistent symptoms. Peri- and post-operative complications were recorded and classified according to the Clavien–Dindo classification [36]. Complications that required subsequent revision of the prosthesis were recorded and classified according to Henderson et al. [20].

Data collected included: patients baseline characteristics, initial diagnosis, type of infection (primary vs. recurrence), number of previous surgeries, ST status, associated ST procedures, DF bone resection length, pathogen, antibiotic therapy duration after first stage, number of repeated first stage, interstage duration, operative time and estimated blood loss at reimplantation, post-operative complications and prosthetic failures unrelated to infection, infectious status at last follow-up (disease free, recurrence of infection), time of infection recurrence, treatment of recurrence.

Quantitative data were summarized by frequencies and percentages for categorical variables, means, standard deviations, and range for continuous variables. A parametric test was used to compare samples in case of continuous variables and normal distribution. The Shapiro–Wilk test was used to verify normal distribution. The Levene’s test was used to analyze homogeneity of the variances. As a parametric test, the two-tailed student’s t-test was used to compare the average of the variables for homoschedastic unpaired groups and the Welch’s t-test for non-homoschedastic unpaired groups. As a non-parametric test, we used the two-tailed Mann–Whitney U test for unpaired groups. Continuity correction was applied in case of discrete distribution. Odds ratios were used to quantify the strength of the association between categorical variables using the χ^2^ statistics (Pearson’s chi-square, Yates’ chi-square, Fisher’s exact test) to establish significance. The Kaplan–Meier method was used to estimate recurrence free survival (RFS) rate. Differences in survival rates were assessed by the log-rank test. Spearman coefficient was used to make correlations. A *p*-value < 0.05 was considered statistically significant. All analyses were completed using the Statistical Package for Social Science (IBM Corp. Released 2013. IBM SPSS Statistics for Windows, Version 26.0. Armonk, NY, USA: IBM Corp.). Graphs were obtained with GraphPad Prism 10 (GraphPad Software, San Diego, CA, USA).

## 3. Results

Sixty patients were recruited for the study. Fifteen patients were excluded: five were lost to follow-up before 2 years, two underwent amputation before reimplantation, seven received knee arthrodesis, and one died of unrelated causes. Therefore, 45 patients were included in the study (Table 1). The mean follow-up time was 43 ± 17.1 months (range, 24–91).

The mean age at presentation was 62.9 ± 15.7 years. The cohort included 24 (53.3%) males and 21 (46.7%) females. The mean CCI was 3.8 ± 2.7. The mean ASA score was 2.7 ± 0.5. The reason for treatment was chronic PJI after TKA in 31 (68.9%) patients, and DF FRI in 14 (31.1%) patients. Patients’ baseline characteristics were homogeneous between PJI and FRI groups. Seventeen (37.8%) patients received a first diagnosis of infection, while 28 (62.2%) patients had been previously treated for chronic infection and relapsed. Of these, 12 (42.9%) patients underwent more than three surgeries for the treatment of the infection. Patients’ characteristics are detailed in Table 1.

All patients presented bone involvement with DF osteomyelitis or severe bone loss and were treated with bone resection at the first stage, after prosthesis or hardware removal. The mean length of resection was 145.4 ± 55 mm (range, 75–290). Twenty-one (46.7%) patients had a fistula pre-operatively. Of these, eight patients (17.8% of all patients) presented severe soft tissue impairment requiring an associated procedure with flap coverage at the first stage. In details, four patients underwent a gastrocnemius rotation flap, three patients a *latissimus dorsi* free flap, and one patient an anterolateral thigh perforator flap. A tibial tubercle osteotomy was performed in eight cases at the first stage. The mean duration of the first stage was 140.3 ± 86.6 min (range, 115–375). The mean estimated peri-operative blood loss was 2.2 ± 1.4 blood units.

The causative organism identified after the first stage were *S. epidermidis* in seven cases, CoNS in seven cases, MSSA in five, MRSA in two, Gram negative bacteria in four cases, *Enterococcus* spp. in three cases, and *C. albicans* in two cases. In three cases, multiple bacteria were isolated from intraoperative tissue samples. In 11 cases, no microorganism was identified at the time of first-stage surgery, but those patients all met the full criteria for the diagnosis of infection.

In 27 (60%) patients, infection was not eradicated after the first-stage surgery and a repeated first-stage revision procedure was required. Of these patients, 14 (51.9%) required more than one repeat of the first stage and, consequently, at least two antibiotic-laden spacers. The treatment of recurrences was found to influence the result of the first stage in terms of infection control. In fact, patients who had been previously surgically treated for infection and relapsed before the first stage had a higher number of repeated first-stage procedures and spacers compared with patients treated for infection for the first time (*p* = 0.003) (Table 1). No other variables were found to increase the risk of repeating the first-stage procedure (Table 1); therefore, multivariate analysis of risk factors was not performed. Seven (15.6%) patients experienced local complications not related to infections after first-stage surgery or repeated first stage, all requiring revision surgery: two spacer breakage/dislocation that required spacer exchange, four hematomas requiring surgical debridement, and one wound dehiscence after a free flap that was successfully treated with superficial debridement and negative-pressure wound therapy (NPWT) before the second-stage surgery.

The mean interstage period was 6.4 ± 4.6 months (range, 1–36). When considering only patients who did not require repeated first stage, the second-stage procedure was performed after a mean of 71.6 ± 18.4 days (range, 28–102). The mean duration of antibiotic therapy after the first stage was 36.9 ± 17.1 days (range, 5–84).

At second-stage surgery with DF SC MP implantation, the mean operative time was 177.5 ± 49.6 min (range, 90–255). The mean estimated peri-operative blood loss was 2.2 ± 1.5 blood units. Tibial tubercle osteotomy was performed in six cases at the second stage.

**Figure 4:**
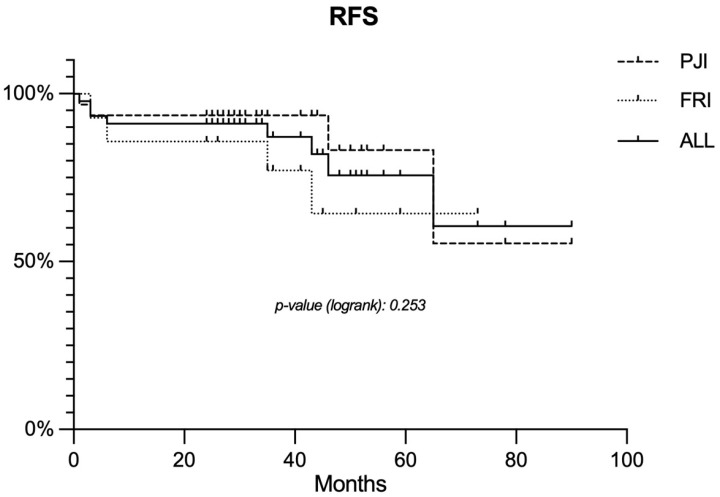
Kaplan–Mayer curve representing the estimated RFS for the entire study cohort and for the PJI and FRI subgroups. Abbreviations: RFS, recurrence free survival; PJI, periprosthetic joint infection; FRI, fracture-related infection.

A total of 16 (35.6%) complications were found related to second-stage surgery. There were eight (17.8%) peri-operative local complications not related to infection: two patella dislocations, two cement extrusions from the tibial diaphysis, three hematomas, and one temporary peroneal nerve palsy. Five complications required revision surgery: two realignment of the extensor apparatus, one removal of the extruded cement, and one surgical debridement for hematoma. There were no aseptic prosthetic failures. Eight (17.8%) patients experienced a recurrent infection during follow-up. Of these, in seven cases at least one of the microorganisms detected after the first stage was isolated at culture analysis of samples from second-stage surgery. In only one case a different pathogen was isolated (MRSA), suggesting a different second infection. The mean time to reinfection was 25.3 ± 25 months (range, 1–65), with no statistically significant differences according to initial diagnosis (PJI vs. FRI). Among possible risk factors for reinfection, only resection length was found to significantly worsen the outcomes in terms of infection control (*p* = 0.031) (Table 1). No other variables were found to increase the risk of reinfection after reimplantation, including the initial diagnosis and the number of repeats of the first-stage procedure (Table 1); therefore, multivariate analysis of risk factors was, again, not performed. Estimated recurrence-free survival (RFS) was 91.1% (93.5% PJI vs. 85.7% FRI) 2 years following MP implantation, and 75.7% (83.2% PJI vs. 64.3% FRI) after 5 years (Figure 4). No statistically relevant differences were found in RFS rates according to the reason for treatment (PJI vs. FRI). To treat reinfection, a total of seven surgical debridement with implant retention (DAIR) and three repeated two-stage procedures were performed. At the last follow-up after recurrence, six patients out of eight presented no signs of infection, while two patients (4.4% of the patients included in the study) underwent above-the-knee amputation (AKA) for uncontrolled infection.

All patients who were infection-free at the last follow-up were able to deambulate autonomously without aids and actively bend the knee.

## 4. Discussion

The vast majority of available literature on non-oncological MPs focuses on either proximal femur implants or included mixed anatomical sites [5,6,11,37,38]. Regarding the DF, indication to MP has been described particularly in TKA periprosthetic fractures, while there is little in the literature with respect to the use of MP to treat knee infections. The present study represents, to our knowledge, the only series of non-oncological DF MP that was homogeneous as far as indication (chronic infections), treatment strategy (two-stage revision surgery with antibiotic-loaded spacers and reimplantation of SC articulated MP) and anatomic site (distal femur).

It is common to observe a severe bone stock deficit following a previous explantation or chronic infection involving the diaphysis of the femur. This is particularly evident in case of PJI recurrences, reported in up to 7–19.7% of cases after surgical treatments during mid-term follow-up of 5 years [39]. Infections following DF fractures are also difficult to treat, both in the case of post-surgical infections after ORIF (reported in 1–2% of cases) [40] and in the case of primary infections from open fractures (ranging from 2.7% to 61%) [41]. Infection-related problems, which may manifest as osteomyelitis or septic non-union, are often combined with those related to bone loss. In contrast to chronic PJIs, whose gold standard treatment is a two-stage revision procedure [29,42], bone defects following severe chronic osteomyelitis and septic non-union may be managed with a wide range of different techniques: distraction osteogenesis, Masquelet-induced membrane technique, allograft reconstructions, and free microvascular bone transfer have largely been used [1,14]. Unfortunately, these techniques generally take a long time with a delayed recovery of functional autonomy [14,43,44]. Furthermore, all these techniques applied to sequelae of DF fractures have serious limitations in case of joint involvement. Finally, given the extreme inhomogeneity of the indications, as well as of the application of the techniques, it is difficult to find reliable evidence on the results of these treatments. In these scenarios, the adoption of a two-stage procedure with bone resection and possible plastic coverage procedures at the first stage in case of ST impairment, followed by MP, is a viable and standardizable option [45,46].

With regard to the results of this study, reinfection rates in this series were found comparable to those described in the literature reporting on staged approaches with implantation of standard primary or revision implants to treat first-episode knee chronic infections at mid-term follow-up (16.2%) [39]. In particular, the reinfection rate in this study was 17.8%, with a mean follow-up of 43 ± 17.1 months, also considering the high amount of already recurrent infections in the study population (62.2%). This appears to be a satisfactory result, as the failure rate of the two-stage procedure to treat chronic infection recurrence at the knee, even without bone resection, is reported to be up to over 70% [47,48,49]. The overall reinfection rate after DF MP in non-oncological settings was reported to be 7.9% in a recent review by Sambri et al. [6]. However, the cohorts in the literature of knee MP in non-oncological cases are very heterogeneous in terms of the reason for treatment, with only a small proportion (around 10%) of cases treated for chronic infection, which obviously significantly lowers the expected rate of septic failures [6]. Moreover, the mean follow-up time of these studies is significantly shorter (36.7 ± 17.8 months) than that of the present study [6].

No significant differences were found in terms of reinfection rates according to initial diagnosis of PJI or FRI. No differences were also found in the timing of reinfection according to initial diagnosis, with a similar time distribution.

In details, the re-infection rate in patients treated for PJI was 12.9%. For PJI managed with a modular MP and a staged approach, Corona et al. described a heterogeneous series including both proximal and distal femur and found 82.8% of patients were infection-free at the last follow-up [50], in alignment with the 87.1% rate in the PJI patient subgroup of this study. In homogenous proximal femur studies, the reported infection eradication rate at the final follow-up ranged from 78% in Sewell et al. [37] and 90.5% in Logoluso et al. [11], to 92% in Dieckmann et al. [38]. Unfortunately, to the best of our knowledge, there is currently only one other study [51] which evaluated the two-stage procedure for chronic PJI of the knee with severe bone loss, using standard DF MP, with no silver coating. This study, which relied on a cohort of 41 patients, reported a reinfection rate of 46.3% with a mean follow-up time of 59 months, resulting in a substantially lower infection control rate than the present study [51]. The large variability in results reported by different studies can be explained by the small number of patients included in each series. All studies agreed, however, that in mid-to-long-term follow-up reinfection rates tended to rise, and the incidence of mechanical complication increased linearly with implant survival time [27].

Regarding the subgroup of patients treated for DF FRI, the reinfection rate was 28.9%, appearing to be considerably lower than that reported in the literature using different techniques, reported to reach 93% [14,43,44].

Although with no statistical significance, the PJI cohort was found to have better infection control than the FRI cohort, as reflected by the lower numbers of infection recurrences: 12.9% and 28.6%, respectively. Although it is not possible to identify a cause-and-effect relationship between the events, it is reasonable to assume that more stringent and rigorous criteria should be applied to decide whether patients with septic non-union may be considered infection-free before proceeding with second-stage surgery, as it is already established clinical practice for PJI.

As a result of univariate analysis for additional potential risk factors for reinfection, out of all the variables considered, only resection length was found to be significantly associated with the risk of reinfection (177.5 ± 50 mm in reinfected cases versus 138.5 ± 54.1 mm in non-reinfected cases). Longer resections are expected to be associated with worse starting conditions, greater surgical exposure, increased blood loss, and a higher risk of wound contamination. However, this finding must be considered incomplete, as the absence of further variables individually affecting the infectious outcome did not allow for multivariate analysis to be performed in order to identify independent risk factors. With the limitations related to the sample size, it can be assumed that the risk of reinfection in highly complex cases depends on several competing factors (e.g., patient-related factors, local soft-tissue conditions, previous treatments, length of resection, intra-operative performance, etc.), which might contribute variably from patient to patient, thus hampering the emergence of single cross-sectional risk factors.

Accordingly, no differences were found in terms of reinfection rate even when segregating patients between first episode infections or recurrent and previously treated infections. However, the latter was found to be a risk factor for the persistence of the infection after the first-stage procedure. Recurrent infections were more difficult to eradicate with a single debridement and resection, and more frequently required multiple debridements. However, after reimplantation, an increase in reinfection occurrences was not observed, either in cases treated for recurrence or in those for which the first stage had to be repeated. This justifies speculation that obtaining apparently complete infection control before reimplantation is the critical factor for treatment success, regardless of the number of previous procedures.

Regarding the soft tissue in the context of a chronic knee infection, as already mentioned, it may be severely damaged, even presenting deep layer exposures or fistulas prior to surgery, or be unable to provide adequate coverage for the spacer or implant after surgery. In this case series, all plastic surgery procedures were performed during the first-stage surgery. Among the included patients, 47% had at least one fistula and 18% required flap coverage. However, neither the presence of fistulas nor the need for associated ST procedures worsened infectious outcomes by acting as a risk factor for reinfection. This suggests, on the one hand, that extensive surgical debridement of all infected tissues, including sinus tracts, provides an adequate outcome if the retained tissues is healthy; on the other hand, it suggests that the use of flap coverage in cases of ST impairment is an effective approach to achieve adequate local vascularity [28].

Saidi et al. suggested that DF MP should be considered a valid treatment option in patients with advanced age and poor bone quality who require early mobilization, but younger patients might not experience comparable benefits, especially in non-oncological settings [45]. A longer life expectancy and higher functional demands in fact inevitably imply a substantial increase in the risk of developing long-term mechanical complications, which is a recognized downside of MP. Such high rates of mechanical complications in MP are explained by the high mechanical stresses on prosthetic components and stems caused by the loss of muscular insertions, and the long lever arm. The rate of aseptic mechanical failure (type II and III failure modes, according to Henderson classifications [20]) of DF MPs in non-oncologic populations is reported to be approximately 5.6% at a mid-term follow-up. In the present study, no patients required revision of the implant due to aseptic loosening or structural failures. In contrast, the total non-infection-related complication rate was 15.6% after the first stage and 17.8% after the second stage, in line with rates reported in the literature [2,13,27,50]. There were only two above-the-knee amputations, both related to re-infection, with an amputation rate of 4.4%, similar to the amputation rate reported in patients undergoing a two-stage procedure for the first episode of knee infection (4%) [52]; the amputation rate for chronic post-traumatic osteomyelitis, on the other hand, is reported to be up to 17% [53,54].

Silver antimicrobial activity relies on several mechanisms. Primarily, it stops the cellular respiratory chain, affecting cell energy generation due to its affinity to the sulfhydryl and thiol groups [21,55,56,57,58]. Additionally, it binds DNA and RNA, disrupting the cellular translation and transcription processes [59], and produces intracellular reactive oxygen species [56]. In the orthopedic field, silver has already found applications in advanced wound dressings and as a coating for MPs, while studies are currently ongoing on its possible use in bone substitutes [22]. The antimicrobial activity of silver coatings can be mediated by the direct release of silver ions into the tissue, or indirectly, through an electrochemical reaction between the different layers of the coating, mediated by silver nanoparticles, as it was the case for the implants employed in this study [57,60]. This is in order to create a toxic environment for microorganisms, hostile to the establishment of bacterial colonies and the formation of biofilms. Several studies have reported so far on the encouraging results of SC implants [42,61,62,63,64]. However, only a few series had previously described the use of SC MP in non-oncologic settings [65,66,67].

The cost of SC MP is higher than that of most alternative techniques for treating severe bone loss, but the time required to reach full weight-bearing is less. Therefore, the higher cost of implants could be probably compensated by the shorter hospital stay and the faster resumption of work productivity in younger patients [68]. Unfortunately, precise cost-effectiveness analyses are difficult to perform for clinical scenarios with limited sample sizes, but it is certainly among the aims of possible future research.

There were significant limitations to this study. Firstly, the design was retrospective, and, therefore, it was subject to inherent limitations and biases, including the lack of quantitative assessment of functional outcomes due to the absence of standardized assessments during follow-ups. Moreover, the absence of a control group did not allow for direct comparisons, which were conducted only as a result of available evidence in the literature, which, furthermore, was not abundant. The sample size was limited, although it was the largest among the studies in the literature on the use of DF MP in patients with chronic knee infections and was the only one reporting data on the use of silver coating in this population. Additionally, the small size of the sample did not allow for further subgroup analyses.

## 5. Conclusions

In the case series presented, articulated megaprosthesis of the distal femur in a two-stage protocol proved to be an effective treatment for chronic knee infection with severe bone loss but a valid extensor apparatus, with a very low amputation rate. However, the total complication rate for this type of surgery is considerable. According to the evidence available in the literature on the treatment of patients with chronic knee infections and severe bone loss, MP appears to provide good results in terms of infection control and total complications, also when compared to other techniques. In case of severe soft tissue impairment, the two-stage protocol is effectively suited for the combination of plastic surgery procedures. Silver coating could contribute to improved treatment results. Comparative studies are required to substantiate the results from this study, with respect to both use of MP in the infectious field and the degree of silver coating influence.

## Figures and Tables

**Table 1:** Patients’ characteristics and univariate analysis of risk factors for repeated first stage and infection recurrence.

Characteristic	Total	Single First-Stage	Repeated First-Stage	*p*-Value	No Reinfection	Reinfection	*p*-Value
Patients, n.	45	18	27	-	37	8	-
Follow-up, months, mean ± SD (range)	43 ± 17.1 (24–91)	39.8 ± 16.3 (26–91)	45.1 ± 17.7 (24–82)	0.316	42.1 ± 17 (24–91)	52.9 ± 13.4 (37–73)	0.099
Age at presentation, y, mean ± SD (range)	62.9 ± 15.7 (28–85)	67.2 ± 14.7 (28–85)	60.1 ± 16 (37–85)	0.102	62.8 ± 16.8 (34–85)	63.3 ± 10.1 (43–75)	0.677
Gender, M/F, n. (%)	24 (53%)/21 (47%)	7 (39%)/11 (61%)	17 (63%)/10 (27%)	0.113	18 (49%)/19 (51%)	6 (75%)/2 (25%)	0.176
BMI, mean ± SD (range)	29.3 ± 5.2 (17.6–45.8)	29 ± 4.6 (23.4–40.4)	29.5 ± 5.7 (17.6–45.8)	0.862	28.7 ± 5.1 (17.6–45.8)	31.8 ± 5.2 (24.1–40.4)	0.432
Diabetes mellitus, yes/no, n.	20/25	8/10	12/15	0.759	16/21	4/4	0.727
Smoking history, yes/no/ex-smoker, n.	11/26/8	4/12/2	7/14/6	0.543	8/22/7	3/4/1	0.627
Alcoholic use, yes/no, n.	6/39	2/16	4/23	0.720	4/33	2/6	0.284
CCI, mean ± SD (range)	3.8 ± 2.7 (0–10)	4.4 ± 2.8 (0–10)	3.5 ± 2.7 (0–10)	0.288	3.9 ± 2.9 (0–10)	3.8 ± 2.3 (1–7)	0.964
ASA score, mean ± SD (range)	2.7 ± 0.5 (1–3)	2.8 ± 0.4 (2–3)	2.6 ± 0.6 (1–3)	0.355	2.7 ± 0.5 (1–3)	2.6 ± 0.4 (1–3)	0.713
Reason to treatment (initial diagnosis): PJI/FRI, n. (%)	31 (69%)/14 (31%)	12 (67%)/6 (33%)	19 (70%)/8 (30%)	0.793	27 (73%)/10 (27%)	4 (50%)/4 (50%)	0.203
Primary infection/recurrence, n. (%)	17 (38%)/28 (62%)	14 (78%)/4 (22%)	13 (35%)/24 (55%)	0.003 *	16 (43%)/21 (57%)	1 (13%)/7 (87%)	0.103
- If recurrence, n of procedures: 1–3/≥4, n.	16/12	1/3	15/9	0.161	13/8	3/4	0.378
Fistula at first-stage, yes/no, n. (%)	21 (47%)/24 (53%)	9 (50%)/9 (50%)	12 (44%)/15 (56%)	0.714	16 (43%)/21 (57%)	5 (63%)/3 (27%)	0.322
Soft tissue injury requiring flap at first-stage, yes/no, n. (%)	8 (18%)/37 (82%)	3 (17%)/15 (83%)	5 (19%)/22 (81%)	0.874	5 (14%)/32 (86%)	1 (13%)/7 (87%)	0.916
Resection length, mm, mean ± SD (range)	145.4 ± 55 (75–290)	144.6 ± 41.8 (90–230)	145.9 ± 63.1 (75–290)	0.826	138.5 ± 54.1 (75–290)	177.5 ± 50 (100–240)	0.031 *
OT at first-stage, min, mean ± SD (range)	140.3 ± 86.6 (115–375)	134.2 ± 91.1 (115–375)	146.2 ± 82.4 (130–40)	0.649	-	-	-
Peri-op EBL at first-stage, BU, mean ± SD (range)	2.2 ± 1.4 (0–9)	2.2 ± 1.9 (0–9)	2.3 ± 1 (1–5)	0.819	-	-	-
Pathogen, type, n.	11 null 7 *S. epidermidis* 7 CoNS 5 MSSA 4 polymicrobial 2 MRSA 4 Gram -^1^ 3 *Enterococcus* spp. 2 *C. albicans*	5 null 2 *S. epidermidis* 2 CoNS 4 MSSA 2 polymicrobial 1 MRSA 2 Gram -	6 null 5 *S. epidermidis* 5 CoNS 1 MSSA 2 polymicrobial 1 MRSA 2 Gram - 3 *Enterococcus* spp. 2 *C. albicans*	-	8 null 6 *S. epidermidis* 5 CoNS 1 MSSA 4 polymicrobial 1 MRSA 4 Gram - 3 *Enterococcus* spp. 2 *C. albicans*	3 null 1 *S. epidermidis* 2 CoNS 1 MSSA 1 MRSA	-
Not infection-related revision after first-stage, yes/no, n.	7/38	2/18	5/27	-	-	-	-
Repeated first-stage, yes/no, n. (%)	27 (60%)/18 (40%)	-	-	-	22 (59%)/15 (41%)	5 (62%)/3 (38%)	0.874
- If repeated, number of spacers: 1/>1, n.	13/14	-	13/14	-	11/11	2/3	0.978
Interstage period, months, mean ± SD (range)	6.4 ± 4.6 (1–36)	-	-	-	6.3 ± 5 (1–36)	6.9 ± 3.2 (2–16)	0.282
Antibiotic-tp after first-stage, days, mean ± SD (range)	36.9 ± 17.1 (5–84)	31.2 ± 18.2 (5–70)	40.7 ± 15.3 (16–84)	0.119	36.8 ± 18.2 (5–84)	37.3 ± 17.1 (5–56)	0.792
OT at second-stage, min, mean ± SD (range)	177.5 ± 49.6 (90–255)	-	-	-	179.4 ± 49.9 (90–250)	168.9 ± 50.3 (115–255)	0.573
Peri-op EBL at second-stage, BU, mean ± SD (range)	2.2 ± 1.5 (0–8)	-	-	-	2.1 ± 1.6 (0–8)	2.6 ± 1.1 (1–5)	0.157
Reinfection, yes/no, n. (%)	8 (18%)/37 (82%)	3 (17%)/15 (83%)	5 (19%)/22 (81%)	0.894	-	-	-
Time to reinfection, months, mean ± SD (range)	25.3 ± 25 (1–65)	-	-	-	-	25.3 ± 25 (1–65)	-
- according to reason to treatment:							
- PJI	28.8 ± 31.9 (1–65)	-	-	-	-	28.8 ± 31.9 (1–65)	-
- FRI	21.8 ± 19.5 (3–43)	-	-	-	-	21.8 ± 19.5 (3–43)	-
Reinfection treatment, type, n.	7 DAIR 3 repeated two-stage	3 DAIR 1 repeated two-stage	4 DAIR 2 repeated two-stage	-	-	-	-
Not infection-related revision after second-stage, yes/no, n.	5/36	-	-	-	4/33	1/7	0.959
AKA amputation, yes/no, n. (%)	2 (4%)/43 (96%)	1 (6%)/17 (94%)	1 (4%)/26 (96%)	-	0/37 (100%)	2 (33%)/6 (67%)	-

* Statistically significant at *p* < 0.05. ^1^ Gram negative bacteria: two *Escherichia coli*, one *Proteus mirabilis*, one *Pseudomonas aeruginosa*, one *Actinomyces bowdenii*. Abbreviations: SD, standard deviation; BMI, body mass index; CCI, Charlson comorbidity index; ASA, American Society of Anaesthesiologists; PJI, periprosthetic joint infection; FRI, fracture-related infection; OT, operative time; EBL, estimated blood loss; BU, blood unit; MRSA, methicillin-resistant *Staphylococcus aureus*; MSSA, methicillin-sensible *Staphylococcus aureus*; CoNS, coagulase-negative staphylococci species; DAIR, debridement, antibiotics, implant retention; AKA, above-the-knee.

## Data Availability

Raw data are available upon reasonable request to the corresponding author.
